# Evaluation of the Effect of a Novel *β3*-Adrenergic Agonist on Choroidal Vascularity

**DOI:** 10.1167/iovs.62.9.17

**Published:** 2021-07-09

**Authors:** Murat Topcuoğlu, Fatih Aslan

**Affiliations:** 1 Alaaddin Keykubat University Education and Research Hospital, Department of Urology, Antalya, Turkey; 2 Alaaddin Keykubat University Education and Research Hospital, Department of Ophthalmology, Antalya, Turkey

**Keywords:** bladder overactivity, choroid, choroidal vascularity index, mirabegron, OCT, retina, β_3_-adrenoceptor agonist

## Abstract

**Purpose:**

To determine the effect of the new *β_3_*-agonist (mirabegron), which is used for overactive bladder (OAB) treatment, on central retinal thickness (CRT) and choroidal vascularity.

**Material and Methods:**

The 26 eyes of 26 cases using 50 mg tablet mirabegron once per day for OAB were included in this prospective case control study. The CRT, choroidal thickness (ChT), and choroidal vascularity were measured at baseline, week 1 (W1), month 1 (M1), month 2 (M2), and month 3 (M3). Subfoveal ChT measurement included the total subfoveal choroidal thickness (SFCT), and the small and large choroidal vessel layer (SCVL and LCVL) thickness. The total choroidal area (TCA), lumen area (LA), stromal area (SA), stroma/lumen ratio, and choroidal vascularity index (CVI) were measured with the Image-J software.

**Results:**

The largest SFCT increase compared to baseline was at M1 (26.8 ± 40.8 µm, *P* = 0.001). The subfoveal SCVL thickness showed a significant decrease at M2 and M3 (−6.0 ± 8.9 µm, *P* = 0.002; −7.8 ± 13.4 µm, *P* = 0.046, respectively). LCVL thickness showed a significant increase at W1, M1, and M2, with the largest at M1. CVI showed a significant increase at M1, M2, and M3 (*P* < 0.05 for all). The TCA, LA, and SA showed a significant increasing trend at all follow-up periods. LA/SA decreased at W1 because of stromal expansion but increased at M3 with more prominent vascular dilatation. CRT values showed no significant change.

**Conclusions:**

Mirabegron had a significant effect on choroidal thickness. Choroidal vascular response is in the form of narrowing in the choriocapillaris and enlargement in the Haller's layer.

Since they have first been cloned in 1989, *β_3_*-adrenergic receptors (AR) have been detected in several human tissues such as adipose tissue, myocardium, gallbladder, central nervous system, blood vessels, urinary system, as well as in the retina.^1^^,^^2^ Under the guidance of recently discovered effects of *β_3_*-adrenoceptors, the role of *β_3_*-agonists in various issues is the subject of continuing research at present, and there is growing interest in β_3_-AR as a novel therapeutic target.^3^^,^^4^ Recently with the support of emerging molecular evidence, the role of *β3*-adrenoceptors as a therapeutic target in ophthalmological diseases has been questioned.^5^ After the first study examining the functional role of the *β_3_*-AR in the eye, which was delivered by Geyer et al.,^6^ the role of *β_3_*-AR related to the control of cell proliferation, migration, invasion and elongation, has been demonstrated in both human retinal and choroidal endothelial cells.^7^^,^^8^

Although various types of *β_3_*-agonists were proposed for the different treatment modalities in myocardium, myometrium, central nervous system, adipose tissue, mirabegron is the only drug which is approved and widely used for the condition of over-active bladder (OAB) in the Unites States and Europe.^9^ OAB is defined as “urgency, with or without urgency incontinence, usually with frequency and nocturia in the absence of either urinary tract infection or proven pathology.”^10^ Mirabegron is a well-tolerated, potent, and selective agent-targeted *β_3_*-AR and first of a new type of pharmacotherapies for the treatment of patients with OAB. It provides an alternative treatment to antimuscarinic agents in the pharmacological management of OAB with a different mechanism and low frequency, as well as low side effects.^11^

Ocular angiogenesis in the posterior segment of the eye likely comes from either choroidal or retinal vascular beds, and several ocular diseases associated with choroidal thickness. Choroid is one of the most vascularized tissues in the body, allowing the outer retina to become avascularized.^12^ Alterations in choroidal thickness (ChT) can cause either hemorrhage and exudation or a decrease in blood flow of the retina. In addition the role of subfoveal choroidal thickness seems to be relevant in the evolution of age-related macular degeneration (AMD).^12^^,^^13^ Thicker choroid has been reported to be a positive predictive factor for treatment response for AMD.^15^ Considering these conditions and the proven role of *β_3_*-ARs in regulating vascular endothelial growth factor level in hypoxic conditions, investigating the changes in vascular bed of the eye after administration of *β_3_-* agonist may be clinically important for diagnosis and treatment.^12^^–^^15^

Clinical ocular effects of mirabegron is uncertain. To the best of our knowledge, there exists no patient-based study in the literature, evaluating the effect of mirabegron on the retinal thickness and choroidal vascularity**.** The aim of the current study was to evaluate the changes in choroidal vascularity and thickness of choroid and retina, 12 weeks after the initiation of the mirabegron treatment and to determine whether it could have a potential promising effect in the treatment of the ocular diseases that are in relation to decreased choroidal blood flow.

## Methods

This prospective study was performed as a joint multi-branch study of ophthalmology and urology departments, between October 2020 and January 2021 under a protocol approved by the Alanya Alaaddin Keykubat University Ethics Committee (24-5/2020), as well as in accordance with the ethical standards laid down in the 1964 Declaration of Helsinki and its later amendments. All patients were given detailed information about the study and provided their written informed consent.

Patients who were admitted to the Alanya Alaaddin Keykubat University Training and Research Hospital's Urology Clinic with complaint of increased voiding frequency, urgency incontinence, or nocturia and diagnosed as OAB were evaluated for the study. In addition to a detailed urological physical examination, medical history, daily voiding frequency, nocturia, and urgency incontinence were obtained. All patients were provided a three-day voiding diary.

Exclusion criteria were previous treatment for OAB or any medications that might affect choroidal thickness, smoking or alcohol abuse, history of coronary artery disease, the presence of hypertension and diabetes mellitus, a history of uveitis, central serous retinopathy, intraocular surgery and a refractive error of ±3 diopters. The criteria used to ensure reliable optical coherence tomography (OCT) image acquisition in the study were that the images had no artifacts, were properly centered, clearly showed distinct retinal layers, and had a signal strength index >45.

Twenty-six patients were enrolled in the study. They were referred to the ophthalmology clinic before the treatment with oral daily mirabegron 50 mg was started. The patients underwent a full ophthalmological examination, including a best corrected visual acuity measurement, biomicroscopic examination, intraocular pressure measurement using Goldmann applanation tonometry, gonioscopy, fundus examination, central corneal thickness measurement (Tonopachy NT-530P; Nidek, San Jose, CA, USA), visual field tests (Octopus; Haag Streit, Mason, OH, USA; 24-2 Tendency-oriented perimetry), axial length measurement (Eye Cubed; Ellex, Adelaide, Australia) and retinal and choroidal evaluation with spectral domain optical coherence tomography (SD-OCT) (RTVue-XR Avanti, Optovue Inc., Fremont, CA, USA). The study consisted of four visits: baseline, first week (W1), first month (M1), second month (M2), and third month (M3). One eye of each subject was randomly selected and analyzed by the computer software.

### OCT Imaging

Choroidal thickness measurements were made in the technique previously reported by Spaide et al.^16^ All of the study eyes were imaged with the RTVue OCT (Optovue Inc., Fremont, CA, USA), a high-speed and high-resolution SD-OCT device with a central wavelength of 840 nm, scan rate of 26,000 A-scans/s, and axial resolution of 5 µm. For the purpose of this study, horizontal B-scan images centered on the fovea were used to perform computed tomography measurements. Each B-scan image is constructed from a number of line scans through the same retinal locations and each line scan consists of 1024 A-scans. With all SD-OCT systems, the signal return from an object is higher when the object is placed nearer the zero delay.

Choroidal thickness was measured perpendicularly from Bruch's membrane, equivalent to the choroid-sclera interface at the fovea, and at two more points located at 1000 µm nasal to the fovea and 1000 µm temporal to the fovea, using the RTVue software (Fig. a). The OCT images obtained within the scope of the study were randomly numbered and recorded by the technician, and the researcher (F.A.) blindly performed the OCT measurements without knowing to whom the images belonged and whether they were made before or after the drug was administered. All basal OCT scans were obtained at the same time of the day (9 to 11 AM) to avoid diurnal fluctuations. Three readings were taken by the grader at different times, and each set of readings was averaged and recorded. The intraobserver reproducibility of the choroidal measurements was evaluated by measuring the interclass correlation coefficient.

**Figure.**
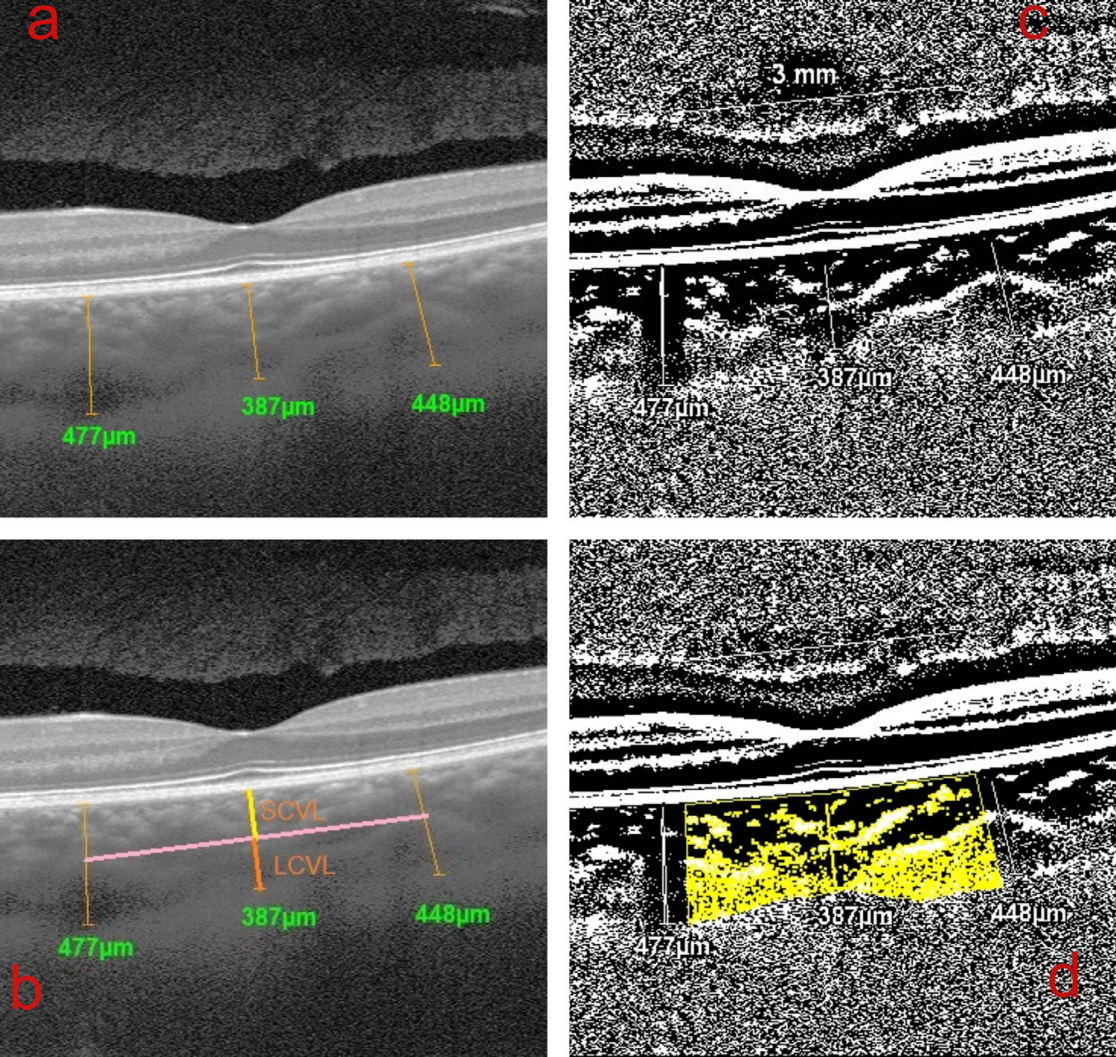
Choroidal thickness (CT) measurement techniques in a sample case. (a) Measurement of total choroidal thickness from 3 points; subfoveal, nasal 1000 µm from center of fovea, 1000 µm temporal from center of fovea. (b) LCVL thickness (Haller's layer) was measured from the inner border of the choroid-scleral junction to the innermost point of the large choroidal vessel at subfoveal location (orange line). Double asterisks indicate large choroidal vessels. Sattler's layer/choriocapillaris layer (SF-SCVL) is the distance from the outer edge of the hyper-reflective retinal pigment epithelium to the orange line and is indicated by the yellow-colored line. Sattler's layer/choriocapillaris layer (SF-SCVL) is separated from the Haller's layer by a purple line. (c) Converted binary image using Image J with the area of interest in the choroid demarcated with a white line. The choroidal area was measured at approximately 3000 µm wide with the margins of 1500 µm nasal and 1500 µm temporal from the foveal center. (d) The color threshold tool is used to select the dark pixels, representing the luminal area. The choroidal vascularity index is computed dividing luminal area by total choroidal area.

Measurement of the large choroidal vessel layer thickness (LCVL) and the Sattler's layer/choriocapillaris thickness (SCVL) at the subfoveal location was undertaken using the method suggested by Branchini et al.^17^ (Fig. b). A large choroidal vessel was defined as a lumen of at least 100 µm in diameter within the choroid.

Binarization was performed with Image-J software (http://fiji.sc./Fiji).^18^ The subfoveal B scan was exported for binarization and choroidal vascularity index (CVI) analysis. We selected 3000 µm wide area with the margins of 1500 µm nasal and 1500 µm temporal from the foveal center. The choroidal area was identified from the retina pigment epithelium to the choroid/sclera junction, and the borders were set manually with the Image-J ROI Manager. Then, the image was converted to 8 bits and adjusted by the Niblack auto local threshold (Fig. c). The total choroidal area (TCA), luminal area (LA), and stromal area (SA) were semiautomatically calculated for all participants. The light pixels were accepted as the SA, and the dark pixels were accepted as the LA (Fig. d).^19^ The CVI was calculated as the ratio between the LA and the TCA.

Retinal thickness in the fovea, parafovea (3.0 mm), and perifoveal areas (6.0 mm) was automatically measured with the device software during the initial and follow-up periods. OCT measurements of the macular area were divided into nine anatomical regions according to Early Treatment Diabetic Retinopathy Study.^20^ Fovea-centered macular measurements with the E-MM5 (0.9 seconds, external 6 × 6 mm grid pattern, internal 4 × 4 mm grid pattern scanning 13 horizontal and 13 vertical lines consisting of 668 A-scans and 8 horizontal and 8 vertical lines, each consisting of 400 A-scans) scanning mode available in the device, were used for the central macular thickness measurements.

### Statistical Analysis

In the descriptive statistics of the data, mean, standard deviation, median lowest, median highest, frequency and ratio values are used. The distribution of variables was measured with the Kolmogorov-Smirnov test. The Mann-Whitney U test was used to analyze quantitative independent data. The paired sample *t* test was used in the analysis of dependent quantitative data. The SPSS 27.0 program was used in the analysis.

## Results

The study group included 26 eyes of 26 OAB patients with a mean age 49.6 ± 13.8 years. Demographic features and baseline ocular parameters are provided in Table 1. Intragrader interclass correlation coefficients and 95% confidence interval (CI) of the grader for baseline, W1, M1, M2 and M3 readings were 0.887 (95% CI, 0.875–0.899), 0.892 (95% CI, 0.821–0.965), 0.912 (95% CI, 0.876–0.948), 0.878 (95% CI, 0.822–0.934), and 0.869 (95% CI, 0.776–0.962), respectively.

**Table 1.** Baseline Demographic and Clinical Characteristics of Patients Included in the Study

Characteristic	OAB Patients
Age (years)	49.6 ± 13.8
Sex (F:M)	17:9
AxL (mm)	21.9 ± 0.9
IOP (mm Hg)	15.8 ± 3.1
BCVA (logMAR)	0.12 ± 0.21
Urgency incontinence episodes (daytime)	2.7 ± 1.1

AxL, axial length; IOP, intraocular pressure; BCVA, best corrected visual acuity; logMAR, logarithm of the minimum angle of resolution.

During the mirabegron treatment, a statistically significant increase was observed in subfoveal choroidal thickness (ChT) for each of the visits, compared to the baseline period (*P* < 0.05 for all terms). The greatest increase in subfoveal ChT compared to the baseline was observed between W1 and M1, with 26.8 ± 40.6 µm in the study group (*P* = 0.014). The amount of change between the M1 and M2 periods was also statistically significant (12.8 ± 27.0 µm, *P* = 0.024). Although increase in temporal 1000 µm ChT was not statistically significant in the W1, M2, and M3 of the study, significant change was observed only in the M1 compared to baseline period (17.3 ± 11.7. µm, *P* = 0.032). Changes in different layers of ChT during treatment period is shown in Table 2.

**Table 2.** Choroidal Thickness Values Of Patients During Treatment Period

	Min-Max	Median	Mean ± SD	*P* ^*^	*P* ^†^
Subfoveal ChT (µm)					
Pretreatment	187–475	310	327.4 ± 86.3		
1^st^ week	181–505	320	338.4 ± 90.1	**0.026**	
1^st^ month	188–561	339	350.2 ± 95.3	**0.021**	0.083
2^nd^ month	191–538	338	336.2 ± 87.6	**0.029**	**0.024**
3^rd^ month	222–468	333.5	339.5 ± 88.1	**0.032**	0.359
SF-SCVL (µm)					
Pretreatment	66–161	110	107.2 ± 28.9		
1^st^ week	66–158	101	106.6 ± 29.1	0.529	
1^st^ month	59–159	105	105.2 ± 27.6	0.925	0.850
2^nd^ month	54–156	99	101.2 ± 26.5	**0.002**	**0.002**
3^rd^ month	64–150	112.5	106.2 ± 28.6	**0.046**	0.240
SF-LCVL (µm)					
Pretreatment	119–414	215	220.2 ± 68.2		
1^st^ week	115–347	231	231.8 ± 63.4	**0.014**	
1^st^ month	129–402	242	245.0 ± 69.7	**0.000**	**0.009**
2^nd^ month	137–382	231	235.0 ± 63.7	**0.001**	0.063
3^rd^ month	148–332	229	233.3 ± 62.3	0.241	0.541
N-ChT (µm)					
Pretreatment	118–484	286	299.7 ± 99.7		
1^st^ week	121–518	311	322.1 ± 97.4	**0.042**	
1^st^ month	125–564	321	327.3 ± 103.3	**0.038**	**0.042**
2^nd^ month	122–534	327	333.2 ± 94.7	**0.046**	**0.034**
3^rd^ month	119–489	333	322.2 ± 112	**0.043**	0.126
T-ChT (µm)					
Pretreatment	187–452	320	310.4 ± 70.2		
1^st^ week	204–494	315	322.0 ± 71.6	0.702	
1^st^ month	212–516	321	336.0 ± 75.7	**0.037**	0.076
2^nd^ month	192–503	322	339.0 ± 72.6	0.981	0.654
3^rd^ month	222–463	318.5	331.0 ± 75.2	0.377	0.945

* Comparison according to the pretreatment period. Significant *P* values are in bold.

† Comparison according to the previous control period. Significant *P* values are in bold.

SF-SCVL, subfoveal small choroidal vessel layer; SF-LCVL, subfoveal large choroidal vessel layer; N-ChT, total choroidal thickness 1 mm from the fovea in the nasal direction; T-ChT, total choroidal thickness 1 mm from the fovea in the temporal direction; SD, standard deviation.

There was no significant change in subfoveal SCVL thickness at the W1 and the M1 compared to the baseline period. Subfoveal SCVL thickness decreased significantly in the M2 and M3 of the study compared to the preop period (−6.0 ± 8.9 µm, *P* = 0.002; −7.8 ± 13.4 µm, *P* = 0.046 respectively). Subfoveal LCVL thickness increased significantly in the W1, M1, and M2 compared to the preoperative period (11.60 ± 36.37 µm, 24.80 ± 44.49 µm, 14.76 ± 31.75 µm, respectively) (Table 2).

TCA, LA, and SA values increased significantly in the W1, M1, M2, and M3 compared to the baseline period. CVI values increased significantly in the M1, M2, and M3 compared to the baseline. LA/SA decreased −0.059 ± 0.047 in the W1 compared to the baseline (*P* < 0.001). LA/SA value increased significantly in the M2 and M3 compared to the baseline period (0.195 ± 0.129 and 0.259 ± 0.120, respectively). Evaluation of choroidal area during treatment period was shown in Table 3.

**Table 3.** Choroidal Vascularity Parameters in Study Cohort

	Min-Max	Median	Mean ± SD	*P* ^*^	*P* ^†^
TCA (mm²)					
Pretreatment	0.35–1.12	0.69	0.68 ± 0.19		
1^st^ week	0.36–1.12	0.69	0.68 ± 0.19	**<0.001**	
1^st^ month	0.37–1.17	0.71	0.70 ± 0.19	**<0.001**	**<0.001**
2^nd^ month	0.38–1.23	0.74	0.76 ± 0.21	**<0.001**	**<0.001**
3^rd^ month	0.51–1.20	0.83	0.82 ± 0.26	**0.005**	**0.005**
LA (mm²)					
Pretreatment	0.16–0.81	0.43	0.43 ± 0.12		
1^st^ week	0.16–0.82	0.43	0.44 ± 0.12	**<0.001**	
1^st^ month	0.17–0.88	0.45	0.46 ± 0.13	**<0.001**	**<0.001**
2^nd^ month	0.18–0.91	0.49	0.51 ± 0.14	**<0.001**	**<0.001**
3^rd^ month	0.24–0.94	0.54	0.55 ± 0.17	**0.005**	**0.005**
SA (mm²)					
Pretreatment	0.08–0.33	0.23	0.24 ± 0.06		
1^st^ week	0.09–0.34	0.24	0.25 ± 0.07	**<0.001**	
1^st^ month	0.09–0.34	0.23	0.24 ± 0.06	**<0.001**	**<0.001**
2^nd^ month	0.09–0.34	0.24	0.25 ± 0.06	**<0.001**	**<0.001**
3^rd^ month	0.13–0.34	0.25	0.26 ± 0.09	**0.002**	**0.005**
CVI (LA/TCA)					
Pretreatment	0.44–0.73	0.65	0.64 ± 0.07		
1^st^ week	0.43–0.73	0.65	0.64 ± 0.07	0.179	
1^st^ month	0.45–0.75	0.66	0.65 ± 0.07	**0.007**	**0.003**
2^nd^ month	0.45–0.75	0.68	0.67 ± 0.07	**0.013**	**0.048**
3^rd^ month	0.47–0.78	0.69	0.67 ± 0.06	**<0.001**	**0.021**
LA/SA					
Pretreatment	1.59–2.82	1.79	1.83 ± 0.29		
1^st^ week	1.55–2.59	1.72	1.76 ± 0.27	**<0.001**	
1^st^ month	1.55–2.81	1.87	1.91 ± 0.30	0.158	**<0.001**
2^nd^ month	1.66–2.79	2.01	2.04 ± 0.30	**<0.001**	**<0.001**
3^rd^ month	1.72–2.55	2.07	2.11 ± 0.22	**<0.001**	**0.007**

* Comparison according to the pretreatment period. Significant *P* values are in bold.

† Comparison according to the previous control period. Significant *P* values are in bold.

SD; standard deviation.

Central retinal thickness (CRT) decreased significantly in the W1 of the treatment. CRT increased significantly in the first month compared to the first week. CRT in the M2 and M3 did not show a significant change compared to the previous visits. CRT did not differ significantly compared to the baseline period in any of the control visits (Table 4). As an outcome of the mirabegron treatment, there was no significant difference in parafoveal and perifoveal thickness, neither in routine visits compared to the baseline period nor in visits compared to the previous visit.

**Table 4.** Retinal Thickness Values of Patients During Treatment Period

	Min-Max	Median	Mean ± SD	*P* ^*^	*P* ^†^
CRT (µm)					
Pretreatment	226–283	247	249.1 ± 13.3		
1^st^ week	224–282	246	247.1 ± 15.1	**0.042**	
1^st^ month	220–282	247	248.6 ± 15.2	0.634	**<0.001**
2^nd^ month	227–282	246	248.9 ± 14.1	0.855	**<0.001**
3^rd^ month	224–264	246.5	246.2 ± 12.0	0.290	**0.005**
Parafoveal RT (µm)					
Pretreatment	295–345	316	316.7 ± 10.7		
1^st^ week	301–346	313	313.8 ± 10.0	0.098	
1^st^ month	299–344	315	315.5 ± 10.1	0.477	0.084
2^nd^ month	300–341	312	314.3 ± 57.8	0.152	0.176
3^rd^ month	303–326	311.5	313.1 ± 7.4	0.887	0.218
Perifoveal RT (µm)					
Pretreatment	275–315	295	295.3 ± 11.9		
1^st^ week	273–315	291	293.2 ± 10.7	0.191	
1^st^ month	275–587	293	305.2 ± 59.4	0.417	0.318
2^nd^ month	276–311	293	293.5 ± 9.5	0.257	0.334
3^rd^ month	277–309	289	293.4 ± 11.8	0.051	0.104

* Comparison according to the pretreatment period. Significant *P* values are in bold.

† Comparison according to the previous control period. Significant *P* values are in bold.

Parafoveal RT, parafoveal retinal thickness; Perifoveal RT, perifoveal retinal thickness; SD, standard deviation.

## Discussion

Choroidal thickness was shown to be mainly affected by the mirabegron treatment in this study, rather than retinal thickness. Subfoveal choroidal thickness showed an increasing trend from baseline, until the third month. We found that the highest increase in ChT was between the first week and the first month (8.5%, *P* = 0.003). In our series, there was no significant change in intraocular pressure during the three-month follow-up. Although a statistically significant decrease in central retinal thickness was shown in the first week, we did not observe any change in parafoveal and perifoveal thickness. Choroidal vessels are innervated by the autonomic nervous system, whereas innervation of the retinal vasculature lack autonomic innervation.^21^ The different effects of mirabegron on the retina and choroid may be due to differences in vascular innervation. It has been shown that in mammals and birds, parasympathetic, sympathetic, as well as sensory fibers and their terminals, tend to be localized to the walls of the arteries and veins of the choroid but not the choriocapillaris.^22^ In this study, we observed a choroidal expansion response after the use of mirabegron to be mostly in Haller's layer. We think that the decrease in thickness of the small choroidal vessel layer compared to the beginning, in the second month and third month, may be caused by the effect of expansion in the Haller's layer. When we evaluated the luminal-stromal component of choroidal expansion, we saw that the enlargement included both compartments, but that the vascular luminal area enlargement was more prominent.

More widespread tissue distribution in the human body as compared to *β_1-2_* AR was reported for *β_3_* in several human tissues, such as the bladder, brain, erectile tissue, adipose tissue, eye, and cardiovascular system; thus *β_3_*-ARs are potential drug targets for a wide range of therapeutic areas.^23^^–^^25^ The *β_3_*-ARs agonists can be divided in two groups, as the first-generation compounds such as BRL37344 and CL316243, and the second-generation compounds such as mirabegron, amibegron, solabegron, ritobegron, and vibegron.^2^^,^^26^ At the time the first generation of compounds was used in human and animal experimental studies, the second generation of compounds was also being used in clinical studies.^9^^,^^27^^,^^28^ Subsequently, using *β_3_*-ARs in the treatment of obesity and type 2 diabetes mellitus has been questioned, and the use of *β_3_*-ARs agonist clinically is largely focused on the treatment of overactive urinary bladder, and mirabegron was approved and widely used in OAB. With *β_3_*-ARs gaining more and more importance as a therapeutic target, the screening for new compounds and new target organs is progressing at a fast pace.^29^^–^^31^

Mirabegron is a potent and selective *β_3_-* adrenergic receptor agonist, approved by the Food and Drug Administration and that has become available for use in the early 2010s the world over. It does not inhibit bladder contractions but facilitates bladder filling and storage via *β_3_*-adrenoceptors on the detrusor muscle.^9^ In our study, mirabegron was given to patients who had not received previous treatment for OAB, and the outcomes were evaluated. Although mirabegron has been licensed and is indicated only in the treatment of OAB, *β_3_*-ARs–related mechanisms that were recently discovered may lead to new therapeutic indications. Since the first support study for the existence and role of the *β_3_*-ARs in the eye, which suggested that this receptor contributes relaxation in the bovine iris sphincter and ciliary, there are very few and only experimental studies about the effects of *β_3_*-ARs and their interactions with pharmacological agents in ophthalmologic area.^6^ We evaluated the effects of a daily dose of 50 mg mirabegron on the choroidal and retinal layers and compared the changes of thickness in these vascular layers in different terms of treatment.

The thickness of choroid and the changes in choroidal layer play an important role in the etiology and pathogenesis of many of the ophthalmologic diseases, such as AMD, diabetic retinopathy, central serous chorioretinopathy and choroidal vasculopathy.^32^^–^^34^ The choroidal layer is particularly important in AMD because abnormalities in the choroidal circulation have been assumed to contribute significantly to the development of AMD.^34^ Age-related macular degeneration is the leading cause of blindness in patients older than 60 years.^35^ The contribution of thinning of the choroid to the etiopathogenesis of AMD was observed in clinical studies.^34^ In a study by Manjunath et al.,^32^ approximately one third of the patients with AMD had thinner than average choroid compared with the age-matched normal volunteers, suggesting a possible role for choroidal thinning in the pathogenesis or progression of AMD.

Focal or diffuse thickening of the choroid, dilated state layer vessels (pachy-veins), thinning in the Sattler layer and choriocapillaris, and choroidal hyperpermeability constitute pachychoroid spectrum diseases (such as central serous chorioretinopathy, polypoidal choroidal vasculopathy) characteristics.^36^ In this study, we found that the choroidal parameters we obtained during the three-month follow-up of mirabegron mimicked the choroidal findings in pachychoroid spectrum diseases. We believe that the significant thinning of the choriocapillaris/Sattler's layer complex (SCVL) in the second- and third-month period and the significant thickening of the Haller's layer (LCVL) in the first- and second-month periods compared to the baseline points toward *β3*-adrenergic blockage as a new treatment target. Therefore it is important to map and observe the choroidal thickness and the vascularity to understand the natural progression of the related diseases.

Although there are no studies on the aqueous humor and vitreous concentrations of mirabegron in humans in the literature, pre-clinical animal studies seem to indicate binding of mirabegron and some of its metabolites to the melanin-containing structures of the eye, as with many adrenoceptor agonists and antagonists. As a result of this binding, radioactivity related to mirabegron has been found to have a very long half-life (157 days) in the eyes of pigmented rats.^37^ This radioactivity has been detected to be high in the ciliary body, choroid and conjunctiva; moderate in the iris, and quite low in the vitreous. Similarly, the eye has been found to be the tissue with the third highest radioactivity amount one week after the oral administration of mirabegron to monkeys.^37^ Dose studies repeated in rats, dogs, and monkeys have not found any ophthalmologic effect or toxicologic effect of the strong melanin binding of mirabegron and its metabolites.^38^ Melanin binding is not an indicator of ocular toxicity by itself, and there are many components that interact strongly with ocular tissues without causing such effects.^39^ The use of mirabegron 100 mg daily for eight weeks has been shown to be safe and well tolerated with regard to intraocular pressure.^40^ Responses of ocular structures at different drug doses will be revealed by future studies, although we observed no ocular or systemic side effects in any patient during three months of mirabegron use at a dose of 50 mg/d.

The most remarkable experimental study about the effects of *β_3_*-agonists in the human donated eye was reported by Steinle et al.^7^ They have aimed to determine which *β*-ARs subtypes are present in human choroidal endothelial cells. Stimulation of the *β_3_*-ARs was achieved by administration of a specific *β_3_*-receptor agonist BRL37344 and statistically significant changes in choroidal endothelial cell proliferation, invasion, and elongation were observed. Observed choroidal changes after stimulation of *β_3_*-ARs that was shown in cultured experimental studies should be supported by clinical studies. Despite limited in vitro studies, there are no clinical studies in the literature regarding the changes caused by an available pharmacological agent in the vascular layers of eye. We observed significant thicker subfoveal choroidal layer in each of the visits during treatment period, compared to baseline values. The most significant increase was observed in the first month, whereas changes in the second and third months of the study seemed stable. Similarly to the subfoveal choroidal layer, a significant increase was observed in the nasal choroidal layer at each visit period, especially in the first and second months. In the temporal layer of choroid, increase in thickness was observed in each of the control visits; additionally the values in the first month and second month were statistically significant.

An earlier study by Steinle et al.^8^ demonstrated for the first time that *β_3_*-ARs exist on human retinal endothelial cells and also suggested that they could regulate migration and play an important role in proliferation, both of which are critical stages of the angiogenesis. Rodent models are currently used to investigate a potential role for *β_3_*-ARs as a therapeutic target in retinopathies. In an animal model by Mori, the role of *β_3_*-ARs in retinal response to hypoxia was investigated in mice's retinal explants.^41^ The crucial role of *β*_3_-ARs in vasodilator responses to adrenaline of retinal arterioles was shown and overall, they showed that *β_3_*-agonist–induced vascular endothelial growth factor release can be a key factor in many hypoxic-ischemic–related retinal diseases, suggesting that the blocking of *β_3_*-ARs may lead to therapeutic tools.^42^ Although a limited number of clinical studies have reported on the vascular response of *β_3_*-ARs in retinal vascular layer, no patient-based trial evaluating the changes in the retinal layer has so far been reported. In our study, unlike the choroidal layer, no significant change was observed in the thickness of the parafoveal and perifoveal retinal regions, at any time of the control visit, despite a significant decrease in the central retina only in the first week of treatment. Longer follow-up time may be required to determine the effect of mirabegron on the retina. There was no difference between male and female patients in terms of changes in the thickness of both the choroid and the retina, during the treatment.

The strength of our study, to the best of our knowledge, is that it is the first study to evaluate the choroidal and retinal evaluation of systemic *β_3_*-agonist agents. This is also the first study in which manual and semiautomatic choroidal measurement were evaluated together. The limitations of our study are the relatively low number of patients, short follow-up period, lack of control group, and the manual measurement technique used for the choroidal thickness. We aimed to decrease our operator-dependent manual measurement thanks to the relatively newly applied semiautomatic choroidal vascularity index.^43^ Choroidal measurements were not corrected for axial length–related magnification because the mean axial length of our study group was shorter than expected for the general population.

In conclusion, the significant increase we observed in the choroidal vascularity parameters after administration of mirabegron, suggests that *β_3_*-ARs may have a role in the treatment of eye diseases associated with the vascular layers. But further experimental and clinical studies, with larger number of patients, are required to support these findings.
